# Genome‐Wide In Vivo RNAi Screening Identifies HOXD4 as a Tumor Metastasis Suppressor in Colorectal Cancer

**DOI:** 10.1002/advs.202520829

**Published:** 2026-06-26

**Authors:** Zhi‐hua Ye, Wen‐jing Luo, Lu Li, Wen‐di Shuai, Ling Zhou, You‐fa Duan, Xue Chen, Jun‐kai Zhang, Wenlin Huang, Ran‐yi Liu

**Affiliations:** ^1^ State Key Laboratory of Oncology in South China Guangdong Provincial Clinical Research Center for Cancer Sun Yat‐sen University Cancer Center Guangzhou Guangdong China; ^2^ Department of Medical Oncology Center Zhongshan City People's Hospital Zhongshan Guangdong China; ^3^ Department of Oncology Qingdao Municipal Hospital Qingdao Shandong China; ^4^ Guiyang Healthcare Vocational University Clinical and Rehabilitation College Guiyang Guizhou China; ^5^ Guangdong Provincial Key Laboratory of Tumor Targeted Drugs & Guangzhou Enterprise Key Laboratory of Gene Medicine Guangzhou Doublle Bioproducts Co. Ltd. Guangzhou Guangdong China

**Keywords:** colorectal cancer, epithelial‐mesenchymal transition, Forkhead box Q1, Homeobox D4, tumor metastasis suppressor

## Abstract

Metastasis remains a major therapeutic challenge in colorectal cancer, highlighting an urgent need to elucidate its underlying molecular mechanisms. In this study, an in vivo screening system integrating genome‐wide short hairpin RNA library and next‐generation sequencing identifies six candidate metastasis suppressors, among which Homeobox D4 (HOXD4) shows the most pronounced effects. Clinicopathological analyses reveal significant HOXD4 downregulation in tumor tissues relative to adjacent normal tissues, with reduced expression strongly correlating with aggressive tumor features. Functional assays demonstrate that HOXD4 depletion enhances migration, invasion, and tumorsphere formation in HCT116 cells, while ectopic HOXD4 overexpression reverses these malignant phenotypes in SW620 cells. Mechanistically, HOXD4 suppresses epithelial‐mesenchymal transition (EMT) by directly binding to the promoter of Forkhead box Q1 (FOXQ1), a key driver of EMT and stemness, and thereby transcriptionally repressing its expression. Immunohistochemistry confirms an inverse correlation between HOXD4 and FOXQ1 expression in clinical specimens. Rescue experiments substantiate that HOXD4 exerts its metastasis‐suppressing functions via FOXQ1 regulation. Collectively, these findings not only establish an efficient platform for screening tumor metastasis suppressors, but also identify HOXD4 as a master transcriptional regulator of the FOXQ1‐EMT axis, providing a promising target for metastasis interception.

## Graphical abstract

Metastasis remains a major challenge in colorectal cancer. Using an in vivo shRNA screening system, this study identifies Homeobox D4 as a key metastasis suppressor. Reduced Homeobox D4 expression is associated with aggressive tumor features. Functional and mechanistic analyses show that it inhibits epithelial‐mesenchymal transition by repressing Forkhead box Q1, highlighting a potential therapeutic target.

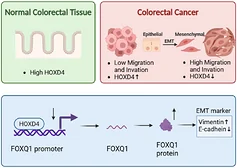

## 1 Introduction

Colorectal cancer (CRC) is one of the most prevalent malignant tumors of the digestive system. According to the World Health Organization, there were over 1.9 million new CRC cases globally in 2022, causing approximately 904 000 deaths [1]. With advances in clinical care, the overall survival rate of CRC patients has improved substantially over the past few decades. Nevertheless, distant metastasis remains the primary driver of poor prognosis. More than 50% of CRC patients eventually develop liver metastases, and these metastases account for more than two‐thirds of all CRC‐related deaths [2, 3]. Accordingly, elucidating the core biological processes and molecular mechanisms driving CRC distant metastasis has emerged as a critical research priority.

Metastasis is a multistep, biologically complex process governed by coordinated regulation of metastasis‐promoting and metastasis‐suppressing genes, enabling tumor cells to escape from the primary lesion and colonize distant organs [4, 5]. Tumor metastasis suppressors (TMSs), typically downregulated in tumor cells [6, 7, 8], differ fundamentally from conventional tumor suppressors: TMSs do not inhibit primary tumor growth, but block metastasis via three core mechanisms—suppressing tumor cell invasiveness, inducing anoikis of disseminated cells during vascular or lymphatic transit, and hindering metastatic colonization at secondary sites [9, 10]. However, research on TMSs lags far behind that of classical tumor suppressors. To date, most studies have investigated TMSs by selecting downregulated genes in tumors and performing individual functional verification, which is time‐consuming and inefficient [11]. In this study, we developed a fast and efficient system for screening TMSs in a nude mouse model using a genome‐wide short hairpin RNA (shRNA) library and next‐generation sequencing, and identified Homeobox D4 (HOXD4) as a novel TMS in CRC.

## 2 Result

### 2.1 Screening for Candidate Tumor Metastasis Suppressors

**FIGURE 1:**
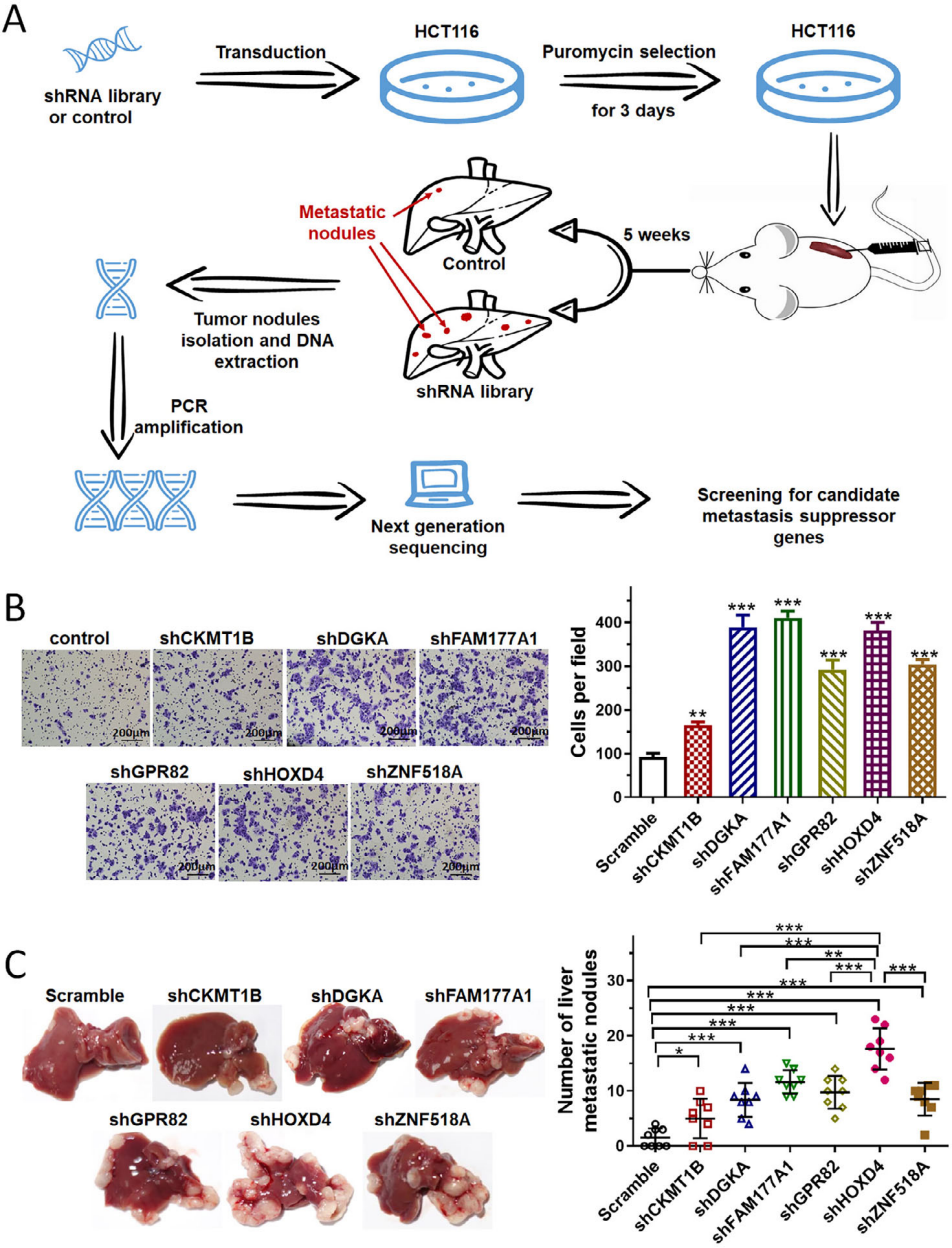
Genome‐wide RNAi screening identifies metastasis‐suppressing genes in colorectal cancer with preliminary functional validation. ‌(A)‌ Schematic of in vivo screening for colorectal cancer metastasis suppressor genes using RNAi library and next‐generation sequencing in a nude mouse splenic injection liver metastasis model. (B)‌ Migration capacity of HCT116 candidate gene knockdown cells assessed by Transwell assay. Left: Representative images; Right: Quantification of migrated cells per field (mean ± SD, *n* = 3 independent experiments). (‌C)‌ In vivo metastatic potential of HCT116 candidate gene knockdown cells. Left: Representative liver macro‐images 5 weeks post‐splenic injection; Right: Metastatic foci quantification. sh, shRNA.

To identify candidate TMSs, we performed a genome‐wide in vivo screening by intrasplenic injection of shRNA library‐transduced HCT116 cells into nude mice (Figure 1A). Representative gross images demonstrated a significantly higher hepatic metastatic burden in the shRNA library group than in control animals (Figure S1A), suggesting that specific shRNAs in the library actively enhance the metastatic potential of HCT116 cells, rather than the difference arising from stochastic effects. Notably, next‐generation sequencing analysis revealed distinct shRNA signatures in individual metastatic nodules, even for multiple lesions isolated from the same animal (Figure S1B,C). This clonal diversity definitively rules out intrahepatic dissemination as the mechanism for metastasis formation in this model.

Following our genome‐wide in vivo screen, seven candidate TMSs were successfully identified: B2M, CKMT1B, DGKA, FAM177A1, GPR82, HOXD4, and ZNF518A (Table S1). Of these, B2M (pool 5) was an essential core component of major histocompatibility complex class I molecules and plays fundamental roles in the systemic regulation of host immune responses [12, 13]. This finding aligns with previous reports showing B2M is downregulated in CRC, where its reduction facilitates tumor immune evasion and correlates with distant metastasis and poor clinical outcomes [14, 15, 16].

For functional validation, we generated stable knockdown HCT116 cell lines targeting the remaining six candidate TMSs (Figure S2A). Subsequent Transwell migration assays (in vitro) and in vivo metastasis assays consistently confirmed that genetic ablation of these candidates significantly enhanced metastatic potential of HCT116 cells both in vitro and in vivo (Figure 1B,C and Figure S2B). These results collectively validate the reliability of our screening system and suggest a low false‐positive rate under our stringent selection criteria.

Among the six candidate TMSs, HOXD4 knockdown in HCT116 cells demonstrated the most robust pro‐metastatic effects, which is supported by two key observations: First, HOXD4‐targeting shRNAs were enriched in metastatic lesions, detected in 4 independent nodules across three distinct mice. This recurrence frequency was the highest observed across all test groups (Figure S1C); Second, nude mice inoculated with HOXD4‐depleted HCT116 cells developed a significantly higher number of metastatic nodules, relative to animals injected with HCT116 cells with knockdown of other candidate genes (*P* < 0.01; Figure 1C and Figure S2B). On the basis of these consistent and reproducible findings, HOXD4 was prioritized for subsequent mechanistic investigations.

### 2.2 HOXD4 is Downregulated in CRC and Serves as an Independent Prognostic Factor for Patient Survival

**FIGURE 2:**
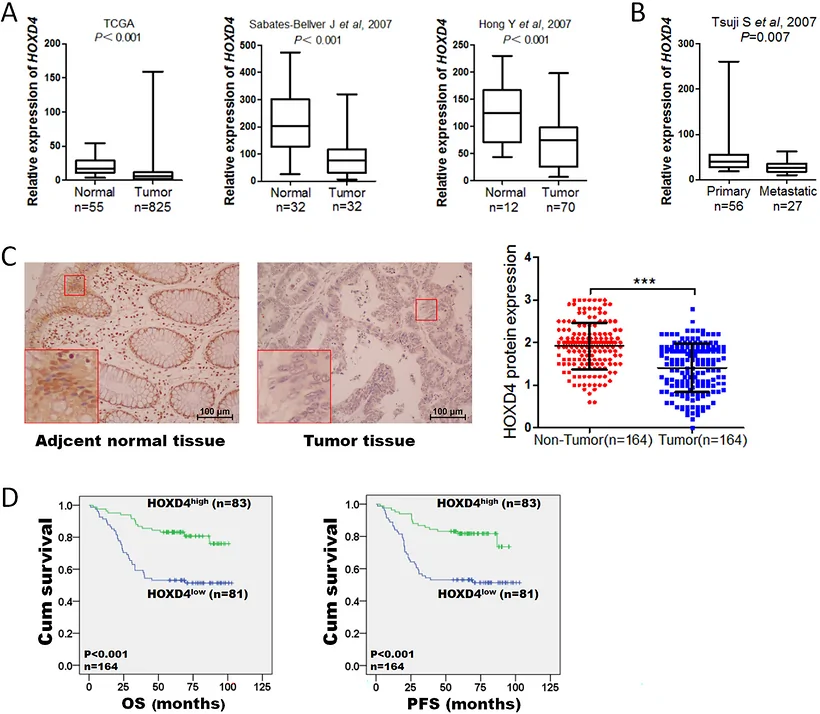
HOXD4 downregulation in CRC predicts poor prognosis. (A) Comparative HOXD4 mRNA expression in CRC vs. adjacent normal tissues analyzed from TCGA cohort and GEO datasets GSE8671 and GSE9348. (B) HOXD4 mRNA levels in primary and metastatic lesions from GEO dataset GSE28702. (C) Immunohistochemical analysis of HOXD4 expression in 164 paired CRC/adjacent normal tissues. Left: Representative images; Right: Statistical analysis of HOXD4 staining scores. (D) Kaplan‐Meier survival analysis of correlation between HOXD4 expression levels and CRC patient prognosis. Left: Overall survival (OS); Right: Progression‐free survival (PFS).

To elucidate the clinical relevance of HOXD4 in human CRC, we first compared HOXD4 expression levels between normal colorectal tissues and primary CRC lesions using three public transcriptomic datasets: two Gene Expression Omnibus (GEO) cohorts (GSE8671 [17], GSE9348 [18]) and CRC project from The Cancer Genome Atlas (TCGA). Concordantly across all three datasets, HOXD4 messenger RNA (mRNA) expression was significantly reduced in primary CRC tissues relative to adjacent non‐tumorous colorectal tissues (Figure 2A). Additional analysis of GSE28702 dataset [19] further revealed that HOXD4 mRNA was more significant downregulated in metastatic lesions relative to primary tumors (Figure 2B). Subsequently, we examined HOXD4 expression at the protein level via immunohistochemical (IHC) staining on an independent clinical cohort comprising 164 histopathologically confirmed CRC tissue specimens. Histochemistry scores (H‐scores) were computed for both tumor tissues and matched adjacent non‐tumorous colorectal tissues to enable quantitative comparison of HOXD4 protein expression. The results showed that HOXD4 protein was predominantly localized to the cell nucleus, and its expression was significantly reduced in CRC tissues relative to matched adjacent non‐tumorous controls (Figure 2C).

Among the 164 CRC specimens included in this study, low HOXD4 expression was detected in 81 cases (49.4%), while high HOXD4 expression was observed in remaining 83 cases (50.6%). To clarify the clinical relevance of HOXD4 in CRC progression, we analyzed the correlation between HOXD4 expression and key clinicopathological characteristics of patients. The results showed that HOXD4 expression was significantly negatively correlated with tumor invasion depth (*P* = 0.045), lymph node metastasis (*P* = 0.007), and distant metastasis (*P* = 0.002; Table 1). Kaplan‐Meier survival analysis revealed that CRC patients with low HOXD4 expression exhibited strikingly poorer overall survival (OS, *P* < 0.001) and progression‐free survival (PFS, *P* < 0.001) compared to those with high HOXD4 expression (Figure 2D). Finally, multivariate Cox regression analysis established HOXD4 as an independent prognostic predictor for both OS (*P =* 0.027, HR = 0.487, 95% CI = 0.258‐0.920) and PFS (*P =* 0.031, HR = 0.495, 95% CI = 0.262‐0.936; Table 2).

**TABLE 1:** Association between HOXD4 levels in colorectal cancer tissues and clinicopathological characteristics.

Variables		No. (%)	HOXD4^Low^	HOXD4^High^	*p* value
Total cases		164 (100)	81 (49.4)	83 (50.6)	
Age(years)	< 60	83 (50.6)	46 (28.0)	37 (22.6)	0.118
	≥ 60	81 (49.4)	35 (21.3)	46 (28.0)	
Gender	Female	73 (44.5)	39 (23.8)	34 (20.7)	0.355
	Male	91 (55.5)	42 (25.6)	49 (29.9)	
Tumor location	Colon	88 (53.7)	43 (26.2)	45 (27.4)	0.885
	Rectum	76 (46.3)	38 (23.2)	38 (23.2)	
Tumor size(cm)	< 5 cm	88 (53.7)	42 (25.6)	46 (28.0)	0.647
	≥ 5 cm	76 (46.3)	39 (23.8)	37 (22.6)	
Tumor invasive depth	T_1‐2_	28 (17.1)	9 (5.5)	19 (11.6)	0.045^a^
	T_3‐4_	136 (82.9)	72 (43.9)	64 (39.0)	
Lymph node status	N_0_	72 (43.9)	27 (16.5)	45 (27.4)	0.070^a^
	N_1‐3_	92 (56.1)	54 (32.9)	38 (23.2)	
Distant metastasis	M_0_	133 (81.1)	58 (35.4)	75 (45.7)	0.002^a^
	M_1_	31 (18.9)	23 (14.0)	8 (4.9)	
TNM stage	I‐II	63 (38.4)	24 (14.6)	39 (23.8)	0.022^a^
	III‐IV	101 (61.6)	57 (34.8)	44 (26.8)	
Preoperative CA199	≤ 35 ng/mL	123 (75.0)	57 (34.8)	66 (40.2)	0.176
	> 35 ng/mL	41 (25.0)	24 (14.6)	17 (10.4)	
Preoperative CEA	≤ 5 ng/mL	99 (61.1)	41 (25.3)	58 (35.8)	0.011^a^
	> 5 ng/mL	63 (38.9)	39 (24.1)	24 (14.8)	

^a^ Statistically significant, *p* < 0.05.

*Note*: According to the 8th Edition of the AJCC Cancer Staging Manual.

**TABLE 2:** Multivariate Cox regression analysis of prognostic factors for patients with colorectal cancer.

	OS		PFS	
Variables	HR (95% CI)	*p* value	HR (95% CI)	*p* value
Tumor Size (cm, <5 vs ≥5)	1.29 (0.70‐2.37)	0.413	1.39 (0.77‐2.53)	0.274
Tumor invasive depth (T1‐2 vs T3‐4)	0.75 (0.27‐2.12)	0.587	0.72 (0.26‐2.02)	0.529
Lymph node status (N0 vs N+)	2.94 (1.32‐6.57)	0.009^a^	3.07 (1.38‐6.82)	0.006^a^
Distant metastasis (M0 vs M1)	5.92 (2.37‐11.78)	<0.001^a^	5.64 (2.87‐11.09)	<0.001^a^
Preoperative CA199 (ng/mL, ≤35 vs >35)	1.47 (0.75‐2.87)	0.263	1.61 (0.84‐3.10)	0.155
Preoperative CEA (ng/mL, ≤5 vs >5)	0.80 (0.41‐1.56)	0.504	0.79 (0.41‐1.52)	0.476
HOXD4 level (low vs High)	0.49 (0.26‐0.92)	0.027^a^	0.50 (0.26‐0.94)	0.031^a^

^a^ Statistically significant, *p* < 0.05.

*Note*: According to the 8th Edition of the AJCC Cancer Staging Manual.

### 2.3 HOXD4 Depletion Promotes CRC Cell Migration and Invasion In Vitro

**FIGURE 3:**
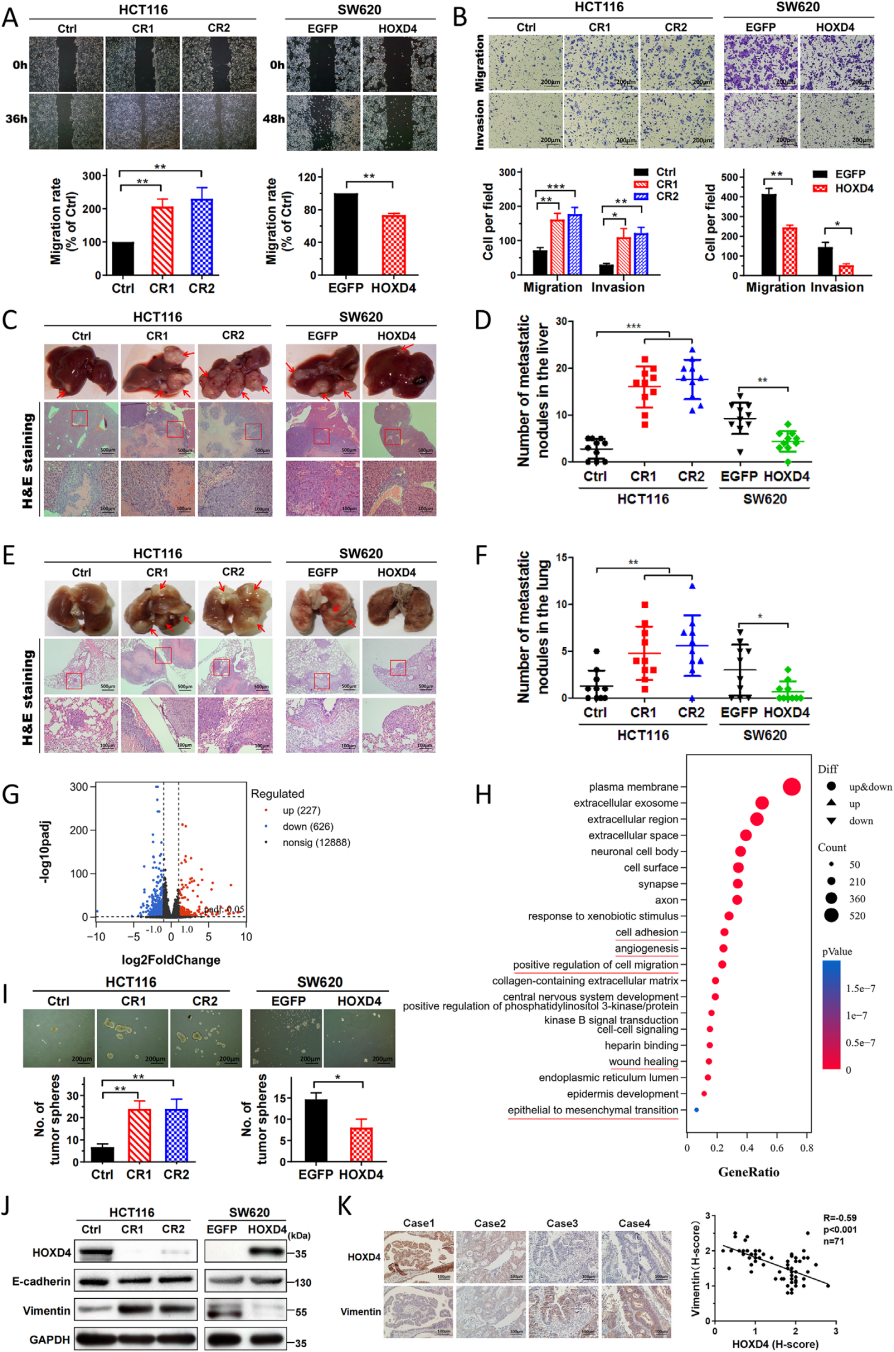
HOXD4 inhibits CRC metastasis by suppressing EMT and tumor sphere formation.‌ (A) Wound healing assay demonstrating the impact of HOXD4 on colorectal cancer cell migration. Microscopic images were captured at 0 h and endpoint, with wound area quantification shown. ‌Top‌: Representative images; ‌Bottom‌: Statistical summary of three independent experiments. (B) Transwell assays evaluating HOXD4's effects on migration/invasion. Crystal violet staining was performed after 24 h incubation, with migrated/invaded cells counted per field. ‌Top‌: Representative images; ‌Bottom‌: Statistical analysis of triplicate experiments. (C, D) Evaluation of HOXD4's impact on CRC liver metastasis in splenic injection model. Nude mice were sacrificed at 6/8 weeks post‐injection of HCT116/SW620 cells, with liver sections HE‐stained. ‌C‌: Representative liver images and histology; ‌D‌: Quantitative analysis of metastatic nodules. (E, F) Assessment of HOXD4's effects on lung metastasis via tail vein injection. Lung sections were HE‐stained at 6/8 weeks. ‌E‌: Representative images; ‌F‌: Metastatic nodule quantification. (G) Volcano plot showing differentially expressed genes (DEGs) between HOXD4 overexpression and control SW620 cells. Upregulated genes (*n* = 227, red) and downregulated genes (*n* = 626, blue) were defined by |log_2_FC|>1 and padj<0.05. (H) Gene Ontology enrichment bubble plot of DEGs. Metastasis‐related pathways are highlighted by red outlines. (I) Sphere formation assay analyzing HOXD4's effect on cancer stemness. Spheres were counted after 7‐day culture. ‌Top‌: Representative images; ‌Bottom‌: Triplicate experiment statistics. (J) Western blot showing HOXD4's regulation of EMT markers (E‐cadherin/Vimentin). (K) IHC analysis of HOXD4/Vimentin expression in 71 primary CRC tissues. ‌Left‌: Representative images; ‌Right‌: Correlation analysis. Ctrl: mock‐transfected control; CR1/CR2: independent HCT116 cell clones with HOXD4 knockout via CRISPR‐Cas9. EGFP, Negative control for overexpression. n.s. not significant; *, *p* < 0.05; **, *p* < 0.01; ***, *p* < 0.001.

To characterize the metastasis‐suppressive function of HOXD4 in CRC, we first profiled its endogenous expression across four CRC cell lines via Western blotting, identifying the highest expression in HCT116 and the lowest in SW620 (Figure S3A). We subsequently generated HOXD4‐knockout HCT116 and HOXD4‐overexpressing SW620 cells, with modification efficiency confirmed (Figure S3B) as detailed in the Materials and Methods section. Wound healing and Transwell functional assays demonstrated that HOXD4 knockout significantly enhanced HCT116 cell migratory and invasion, whereas HOXD4 overexpression markedly reduced these pro‐metastatic properties in SW620 cells (Figure 3A,B).

### 2.4 HOXD4 Depletion Promotes CRC Metastasis in Xenograft Mouse Model

To assess metastatic potential in vivo, we inoculated nude mice with control (Ctrl) and HOXD4‐knockout (CR1/CR2) HCT116 cells via intrasplenic injection (liver metastasis model) or tail vein injection (lung metastasis model). Six weeks post‐injection, histological analysis demonstrated that HOXD4‐deficient HCT116 cells developed substantially larger and more numerous metastatic nodules than controls in both liver (Figure 3C,D) and lung (Figure 3E,F) evidenced by both gross morphology and H&E‐stained tissue sections.

Conversely, parallel experiments in SW620 cells revealed that HOXD4 overexpression (SW620‐HOXD4) markedly reduced metastatic capacity: eight weeks post‐injection, SW620‐HOXD4 formed significantly smaller and fewer nodules than SW620‐EGFP control in both liver and lung metastasis models (Figure 3C–F).

To exclude proliferation as a confounding variable for metastasis‌, 3‐(4,5‐dimethylthiazol‐2‐yl)‐2,5‐diphenyltetrazolium bromide (MTT) and colony formation assays confirmed that HOXD4 modulation ‌did not affect CRC cell proliferation rates‌ (Figure S4). ‌Collectively, these in vitro and in vivo findings demonstrate that HOXD4 functions as a metastasis‐suppressor in CRC.

### 2.5 HOXD4 Inhibits Anchorage‐Independent Growth and Epithelial‐Mesenchymal Transition in CRC

To delineate the molecular mechanisms underlying HOXD4's metastasis‐suppressive function, we performed RNA sequencing (RNA‐seq) analysis on HOXD4‐overexpressing SW620 cells. Differential expression analysis revealed a global predominance of downregulated genes (Figure 3G), indicating that HOXD4 acts primarily as a transcriptional repressor in CRC cells—an expression profile consistent with its proposed role as a metastasis suppressor. Gene Ontology (GO) enrichment analyses confirmed that differentially expressed genes (DEGs) were significantly enriched in metastasis‐related biological processes (Figure 3H), and the vast majority of genes within these enriched pathways were downregulated (Figure S5). Collectively, these data indicate that HOXD4 overexpression globally suppresses pro‐metastatic transcriptional programs, thus attenuating the metastatic capacity of CRC cells.

To investigate tumor cell survival capacity following detachment, we systematically evaluated anchorage‐independent growth potential and epithelial‐mesenchymal transition (EMT) features in CRC cells. Sphere formation assays demonstrated that HOXD4 depletion in HCT116 cells significantly enhanced sphere‐forming efficiency (2.6‐fold increase vs. controls, *P* < 0.01), whereas HOXD4 overexpression in SW620 cells markedly reduced this capacity (45.5% reduction, *P* < 0.05; Figure 3I).

Morphometric analysis revealed distinct phenotypic differences: HOXD4‐overexpressing cells retained a compact epithelial morphology with prominent cell‐cell junctions, whereas HOXD4‐deficient cells exhibited an elongated mesenchymal morphology with reduced intercellular contacts (Figure S6). These morphological changes were further corroborated by molecular profiling, with decreased Vimentin expression detected in HOXD4‐overexpressing cells and reciprocal upregulation in HOXD4‐knockout cells (Figure 3J). Clinical correlation analysis of 71 CRC specimens confirmed an inverse relationship between HOXD4 and Vimentin expression (Pearson r = ‐0.59, *P* < 0.001; Figure 3K).

Collectively, these data demonstrate that HOXD4 acts as an EMT suppressor in CRC—EMT is a core metastatic process that endows cancer cells with invasive and stem‐like properties—by directly repressing Vimentin expression [20, 21, 22].

### 2.6 HOXD4 Transcriptionally Represses FOXQ1 Expression in CRC

**FIGURE 4:**
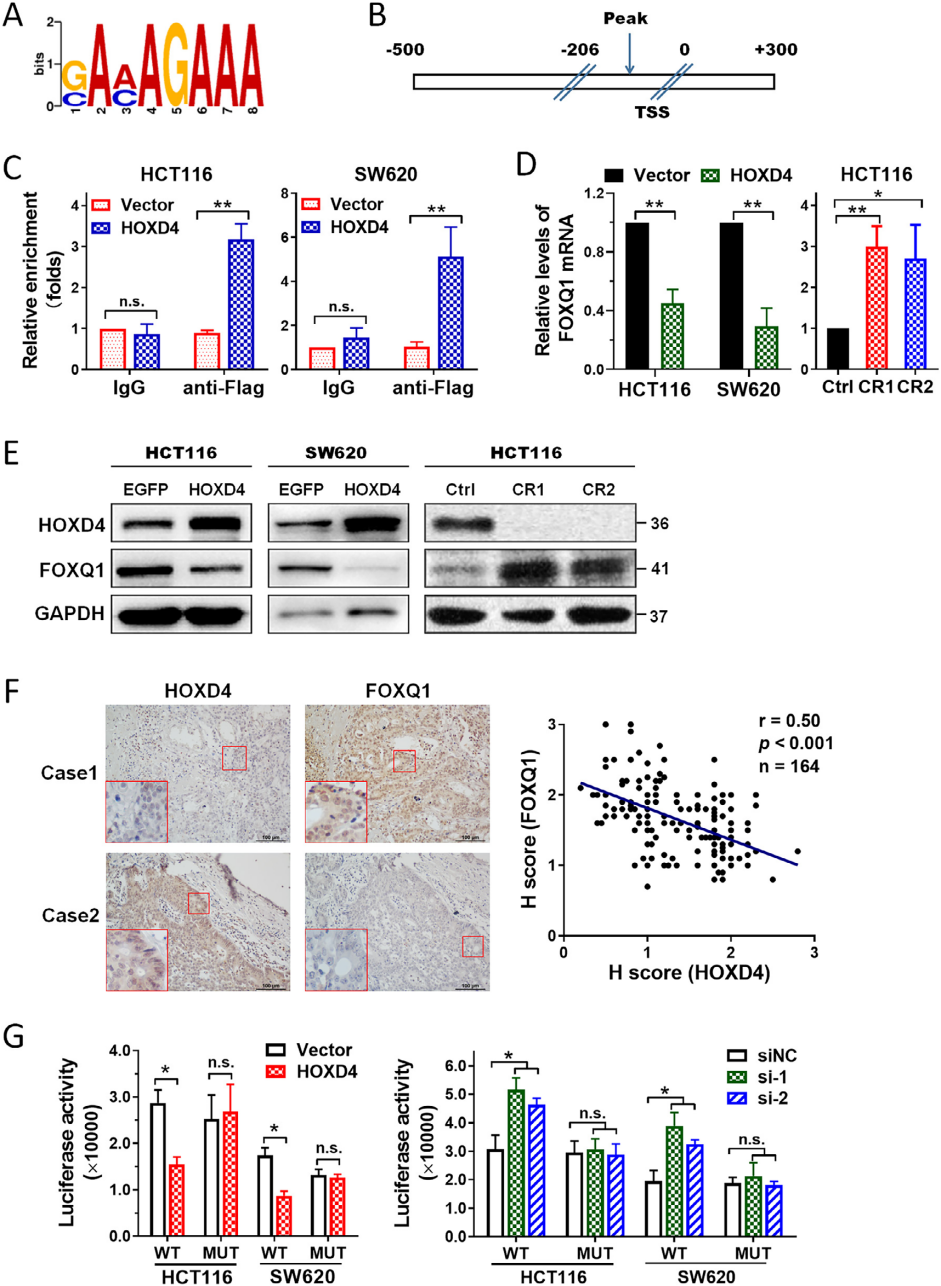
HOXD4 transcriptionally represses FOXQ1 expression in CRC through direct binding to the FOXQ1 promoter.‌ (A) Identification of HOXD4 genomic binding targets via ChIP‐seq, with prediction of the most probable binding motif using DREME analysis. (B) Schematic representation of the HOXD4‐binding region in the FOXQ1 promoter. (C) ChIP‐qPCR validation of FOXQ1 promoter enrichment in ChIP fragments from CRC cells (Statistical summary of three independent experiments). (D) RT‐qPCR analysis of HOXD4's effect on FOXQ1 mRNA levels in CRC cells (Triplicate experiment statistics). (E) Western blot detection of HOXD4's impact on FOXQ1 protein expression in CRC cells (GAPDH as loading control). (F) Immunohistochemical analysis of HOXD4/FOXQ1 expression in 164 primary CRC tissues. Left: Representative images; Right: Correlation analysis. (G) Luciferase reporter assay to evaluate HOXD4's effect on FOXQ1 promoter activity in CRC cells (Statistical analysis of triplicate experiments). Ctrl: Mock‐transfected control; CR1/CR2: Independent HCT116 clones with HOXD4 knockout via CRISPR‐Cas9; EGFP: Negative control for overexpression; siNC: Negative control siRNA; si‐1/si‐2: Independent HOXD4 siRNAs; WT/MUT: Wild‐type/mutant FOXQ1 promoter. *, *p* < 0.05; ‌**, *p* < 0.01; n.s., not significant.

To delineate the molecular mechanism underlying HOXD4‐mediated regulation of CRC metastasis, we performed chromatin immunoprecipitation sequencing (ChIP‐seq) to profile genome‐wide HOXD4 binding. Consistent with its canonical role as a transcription factor, HOXD4 was found to preferentially occupy gene promoter regions. Motif enrichment analysis identified 5’‐SAMAGAAA‐3’ as the top‐scoring consensus binding sequence for HOXD4 (Figure 4A and Figure S7A).

Notably, Forkhead box Q1 (FOXQ1), a well‐characterized oncogene previously reported to drive EMT and distant metastasis in CRC [23], was retrieved among the high‐confidence candidate target genes of HOXD4 in our ChIP‐seq dataset. Given its well‐established oncogenic role in CRC progression, we prioritized FOXQ1 for subsequent mechanistic validation. Detailed genomic mapping of the HOXD4 binding peak revealed an enrichment peak located 122 bp upstream of the FOXQ1 transcription start site (TSS) (Figure 4B and Figure S7B). Subsequent locus specific ChIP‐PCR assay independently confirmed the specific binding of HOXD4 to the region spanning ‐206 bp to 0 bp relative to the FOXQ1 TSS (Figure 4C).

To determine whether HOXD4 regulates FOXQ1 expression in CRC cells, we performed reverse transcription quantitative PCR (RT‐qPCR) and Western blot analysis. In HCT116 cells, HOXD4 overexpression significantly reduced both FOXQ1 mRNA and protein levels, whereas HOXD4 knockout upregulated FOXQ1 expression (Figure 4D,E). IHC analysis of 164 CRC tissues further confirmed an inverse correlation between HOXD4 and FOXQ1 protein levels (Figure 4F).

To mechanistically validate this regulatory relationship, we generated luciferase reporter systems for the FOXQ1 promoter in HCT116 and SW620 cells via lentiviral transduction. Promoter activity assays showed that HOXD4 overexpression significantly inhibited luciferase expression driven by the wild‐type FOXQ1 promoter, whereas HOXD4 knockdown enhanced promoter activity. Importantly, this regulatory effect was completely abolished by deletion of the ‐153 to ‐92 bp region in the FOXQ1 promoter (mutant reporter, Figure 4G). Collectively, these data indicate that HOXD4 directly represses FOXQ1 transcription through specific binding.

Subsequent assay for transposase‐accessible chromatin with high‐throughput sequencing (ATAC‐seq) revealed no significant changes in chromatin accessibility at the FOXQ1 promoter following HOXD4 overexpression (Figure S7C). Notably, the ‐153 to ‐92 bp FOXQ1 promoter fragment to exhibited a constitutively open chromatin state in both control and HOXD4‐overexpressing cells, indicating that this region is accessible prior to HOXD4 perturbation.

### 2.7 HOXD4 Modulates CRC Metastasis via FOXQ1

**FIGURE 5:**
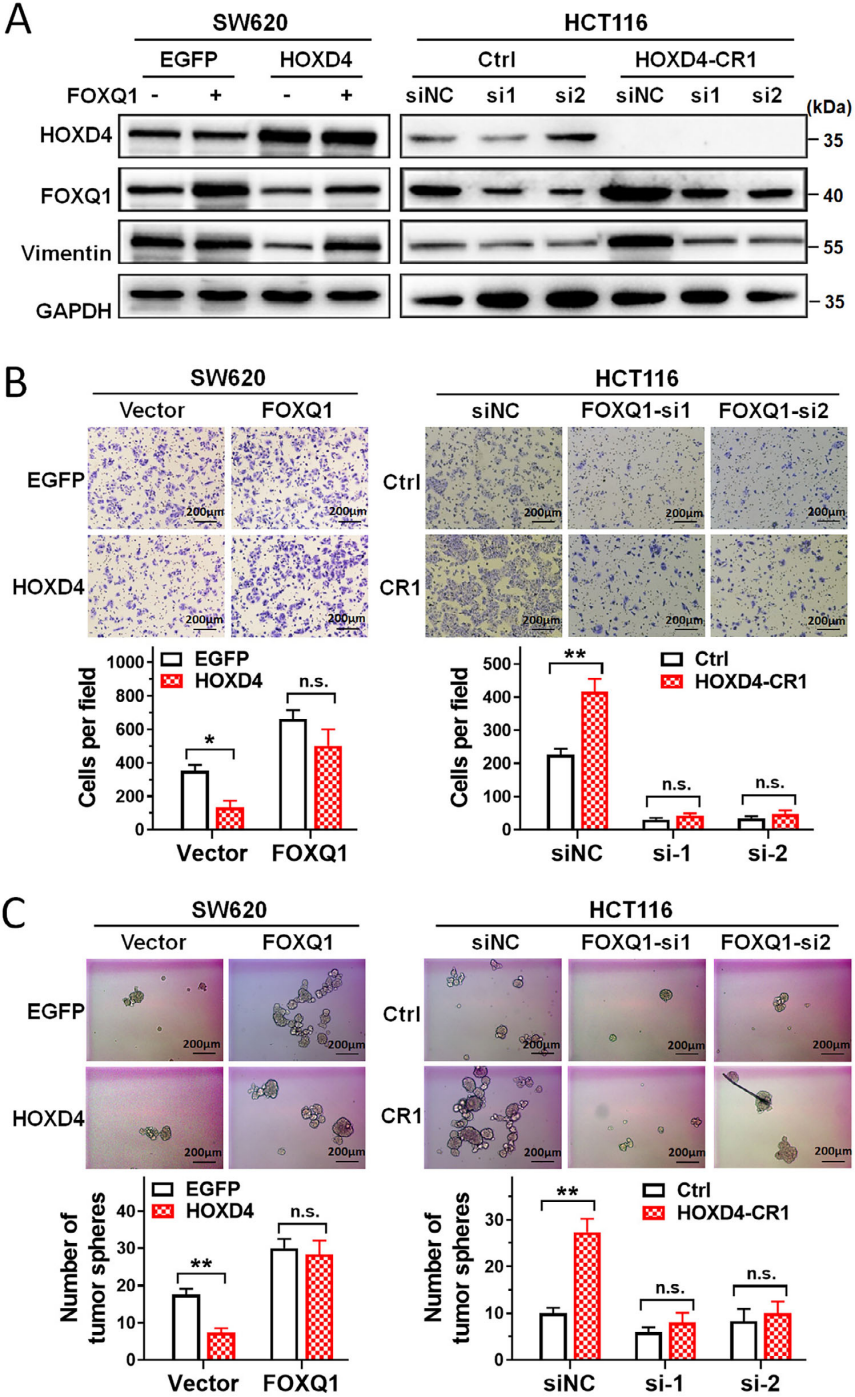
Rescue experiments demonstrating that HOXD4 regulates CRC metastasis through FOXQ1.‌ (‌A)‌ Western blot analysis of HOXD4's effects on Vimentin expression in CRC cells with ‌FOXQ1 knockdown or overexpression (GAPDH as loading control). (‌B)‌ Transwell migration assay assessing HOXD4's effects on CRC cell migration in the context of ‌FOXQ1 knockdown or overexpression‌. Top: Rpresentative images; Bottom: Statistical summary of three independent experiments. (‌C)‌ Sphere formation assay evaluating HOXD4's effects on the non‐adherent growth of CRC cells under ‌FOXQ1 knockdown or overexpression conditions‌. Top: Rpresentative images; Bottom: Statistical analysis of triplicate experiments. Ctrl: Mock‐transfected control; CR1: a HCT116 clone with HOXD4 knockout; EGFP: Negative control for overexpression; siNC: Negative control siRNA; si‐1/si‐2: Independent FOXQ1 siRNAs. *, *p* < 0.05; ‌**, *p* < 0.01; n.s., not significant.

To further confirm that HOXD4 suppresses CRC metastasis via transcriptional repression of FOXQ1, we performed a series of functional rescue experiments incorporating Western blot, Transwell migration, and sphere formation assays. In SW620 cells, HOXD4 overexpression reduced Vimentin expression and significantly suppressed both cell migration and sphere formation efficiency; these phenotypic effects were markedly attenuated by concomitant exogenous FOXQ1 overexpression. Conversely, small interfering RNA (siRNA)‐mediated knockdown of endogenous FOXQ1 partially reversed the pro‐metastatic phenotype induced by HOXD4 depletion in HCT116 cells (Figure 5). Collectively, these data demonstrate that HOXD4 exerts its anti‐metastatic activity in CRC primarily through direct transcriptional inhibition of FOXQ1.

## 3 Discussion

Homeobox genes encode evolutionarily conserved transcription factors with critical roles in embryonic development and cellular differentiation [24, 25]. Although homeobox genes have been well‐documented as contributors to cancer progression [26, 27, 28], the biological role of HOXD4 remains poorly defined. Previous studies have associated HOXD4 overexpression with unfavorable prognosis in ovarian cancer, gastric adenocarcinoma, and glioma [29, 30, 31, 32], but the underlying molecular mechanisms have not been systematically investigated. This study identified HOXD4 as a novel TMS: HOXD4 inhibits CRC cell migration and invasion in vitro, and suppresses liver and lung metastasis in nude mice; HOXD4 is frequently downregulated in tumor tissues, which strongly correlates with advanced disease progression including deeper invasion, lymph node/distant metastasis, and unfavorable patient outcomes. These findings suggest that HOXD4 may serve as a promising independent prognostic biomarker and potential therapeutic target for CRC metastasis.

Distant metastasis is a multistep process encompassing local invasion, circulatory survival (anoikis resistance), and metastatic colonization [33, 34]. Metastatic potential is collectively marked by EMT [35] and non‐adherent spheroid formation capacity [36]. Vimentin, a core EMT regulator [37], coordinates expression of key EMT transcription factors (Snail, Slug, Twist, ZEB1/2) and epigenetic modifiers [38, 39, 40, 41], while driving cancer stemness via self‐renewal activation and differentiation suppression [42, 43, 44]. Our findings precisely corroborate these mechanisms: HOXD4 inhibits distant CRC metastasis through dual suppression of EMT and spheroid formation—two well‐established hallmarks of metastatic potential.

Further, our study reveals a novel regulatory mechanism by which HOXD4 transcriptionally suppresses FOXQ1 expression via direct binding to the ‐153 to ‐92 bp region of its promoter. As an oncogenic FOX family transcription factor, FOXQ1 is aberrantly overexpressed across multiple malignancies [45, 46, 47], and serves as a master regulator of EMT through direct modulation of EMT‐associated transcriptional networks. It drives cadherin switching and upregulates EMT markers including Vimentin via TGF‐β/Wnt‐mediated activation of Snail/Zeb2/Sox12 transcriptional axis [48, 49]. Notably, ATAC‐seq analysis revealed that the FOXQ1 promoter region (‐153 to ‐92 bp) resides in a constitutively open chromatin state, and its accessibility remains unchanged following HOXD4 overexpression. This finding suggests that HOXD4‐mediated transcriptional repression of FOXQ1 occurs independently of large‐scale chromatin accessibility remodeling. Two potential models could explain this observation: HOXD4 may recruit repressive chromatin modifiers such as Polycomb Repressive Complex 2 (PRC2), which mediates H3K27me3 deposition without altering overall DNA accessibility to Tn5 transposase; alternatively HOXD4 binding may cause steric hindrance that blocks the transcriptional pre‐initiation complex. This “open yet repressed” chromatin conformation at the HOXD4 locus aligns with the well‐characterized context‐dependent repressive activity of Homeobox family transcription factors [50] and further consolidates the mechanistic framework underlying the HOXD4–FOXQ1 regulatory axis.

The clinical implications of our study are twofold. First, the identified HOXD4→FOXQ1→Vimentin→EMT regulatory axis not only improves our understanding of CRC metastasis but also highlights HOXD4 as both a promising prognostic biomarker and a high‐potential therapeutic target. Second, although the clinical utility of TMSs remains limited at present [51, 52], emerging strategies to restore TMS function—including gene therapy, epigenetic modulation, and pathway‐targeted interventions [53]—offer a robust translational framework for clinical development. Approaches targeting either HOXD4 expression restoration or its downstream effectors such as FOXQ1 inhibitors hold particular promise, and can be incorporated into existing combination therapies for metastatic CRC. To realize this therapeutic potential, future work should focus on three key directions: (1) delineating the upstream regulators of HOXD4 expression; (2) developing small‐molecule modulators targeting the HOXD4‐FOXQ1 axis; and (3) characterizing synergistic interactions with conventional chemotherapeutics. Additionally, the exact nucleotide‐level binding determinants of HOXD4 within the FOXQ1 promoter remain to be fully characterized and represent an important future direction. These studies will ultimately facilitate the development of precision medicine strategies for clinical management of metastatic CRC. Although our findings show promising clinical implications, they are primarily based on two‐dimensional CRC cell models that do not fully recapitulate the tumor microenvironment. Nonetheless, our results are supported by in vivo evidence, and future studies incorporating organoid models will further strengthen their physiological relevance.

## 4 Materials and Methods

### 4.1 Cell Lines, siRNAs, Plasmids, and Transfections

293T and CRC cell lines were obtained from the American Type Culture Collection (ATCC) and were cultured according to the ATCC instructions. SiRNAs (their sequences are listed in Table S1) were synthesized by GenePharma (Suzhou, Jiangsu, China). pENTER‐HOXD4‐Flag was purchased from Vigene Biosciences (Shandong, China). FOXQ1 complementary DNA (cDNA) was cloned into the plasmid pcDNA3.1 with a Flag tag. Plasmids and siRNAs were transfected using Lipofectamine 2000 (Invitrogen, Carlsbad, CA, USA) according to the manufacturer's instructions.

### 4.2 Animal Model

Female BALB/c nude mice (4‐5 weeks old, 15–18g) were purchased from the SLRC laboratory animal (Shanghai, China). For the in vivo metastasis model, the mice were anesthetized with isoflurane and laparotomized to pull the spleen out of the abdominal cavity. A total of 1 × 10^6^ cells were slowly injected into the spleen. In addition, 1 × 10^6^ cells were injected into the tail vein of mice to evaluate metastatic potential. Each groups contained 10 mice. After 6 weeks (HCT116) or 8 weeks (SW620), the mice were sacrificed and the livers or lungs were removed for pathological examination as described previously [54]. For the in vivo RNAi screening, nude mice were sacrificed 5 weeks after injection. Animal experiments were approved by the Sun Yat‐sen University Cancer Center Institutional Animal Care and Usage Committee (No. L102012018006K).

### 4.3 Genome‐Wide In Vivo RNAi Screening for CRC Metastasis Suppressor Genes

Genome‐wide in vivo RNAi screening was performed using the LentiPlex^TM^ human whole‐genome shRNA library targeting 15 000 human genes (with an average of 5 shRNAs per target gene) (SHPH01, Sigma‐Aldrich, St. Louis, MO, USA) according to the manufacturer's instructions. The CRC HCT116 cells were transduced with each subpool (subpools 1–10) of the shRNA library, respectively, at a multiplicity of infection (MOI) of 0.5 and selected with puromycin for 72 h before being injected into the spleens of nude mice (5‐9 mice per subpool). Five weeks later, liver metastases were dissected for genomic DNA extraction. Following PCR amplification and next‐generation sequencing analysis, potential candidate genes were systematically identified through bioinformatic alignment of shRNA sequences with their corresponding target genes in the reference database (Figure 1A). The selection criteria were rigorously applied as follows: (i) Biological relevance requirement: at least one specific shRNA sequence needed to be consistently detected in ≥2 distinct metastatic lesions, with its read count exceeding 5% of the total shRNA reads in each sample; (ii) Clinical correlation requirement: independent validation using ≥2 independent datasets from the Oncomine platform (threshold: *P* < 0.05, fold change >1.5) demonstrating significant downregulation of the candidate gene in CRC tissues compared to normal colorectal mucosa.

### 4.4 Bioinformatics Analysis of Gene Expression in CRC

GEO datasets GSE8671 [17] and GSE9348 [18] and the TCGA database for the CRC (COAD and READ) project were used to compare the expression of HOXD4 in CRC tissues with that in control non‐tumorous colorectal tissues, while the dataset GSE28702 [19] was used to analyze the expression of HOXD4 in primary and metastatic lesions.

### 4.5 Tissue Samples and Immunohistochemical Staining

Surgical specimens of paired tumor and adjacent non‐tumorous colorectal tissue were collected from 164 CRC patients at Sun Yat‐sen University Cancer Center (SYSUCC, Guangzhou, China) who did not receive neoadjuvant therapy prior to surgery. The specimens were paraffin‐embedded and analyzed for the expression of HOXD4, Vimentin and FOXQ1 by IHC staining (anti‐HOXD4 and anti‐FOXQ1: #HPA070349 and #AV39755, Sigma–Aldrich; anti‐Vimentin: #60300‐1, Proteintech, Wuhan, China). The expression levels are presented as the H score [55], which was independently evaluated by two pathologists. An H score of 1.45 was determined as the cut‐off value in this study to distinguish between low or high HOXD4 expressions using the receiver operating characteristic (ROC) curve analysis. The study protocol was approved by the institutional review board and human ethics committee of SYSUCC (No. GZR2018‐164).

### 4.6 The Generation of Stable Cell Lines

HOXD4 cDNA was cloned into the pCDH‐EF1‐MCS‐T2A‐Puro vector (System Biosciences, Palo Alto, CA, USA), and the construct was then packaged in 293T cells to generate lentiviruses that were used to generate cell lines with stable overexpression of HOXD4 (EGFP was used as a negative control) [56]. Small guide RNAs (sgRNAs) targeting HOXD4 and the negative control sgRNA (Table S1) were cloned into lentiCRISPR v2 [57], a gift from Dr. Feng Zhang of the Massachusetts Institute of Technology, for the generation of HOXD4 knockout cell lines; monoclonal cell lines with HOXD4 knockout were generated by limiting dilution and verified by PCR/Sanger sequencing.

### 4.7 Wound Healing Assays

Cell‐free gaps, mocking wounds, were created by scratching confluent monolayers in a 6‐well plate using a 100µl pipette tip. The plate was washed with phosphate‐buffered saline (PBS), and then incubated in serum‐free medium. Wound healing was monitored and photographed under a microscope every 12h from 0 to 48h post‐scratch [58]. The Image‐J software was used to analyze and calculate for the rate of cell migration [59].

### 4.8 Transwell Migration and Invasion Assays

For migration or invasion assays, 1 × 10^6^ cells suspended in serum‐free medium were seeded into the upper compartment of 8‐µm pore Boyden chambers (BD Falcon, San Jose, CA, USA) with or without Matrigel (#356234, BD). The chambers were then placed in 24‐well plates containing medium supplemented with 10% FBS. After 24 h of incubation, non‐migrated/invaded cells on the upper membrane surface were removed, and the cells that traversed the membrane were fixed and stained with 0.5% crystal violet in methanol. Five random fields per membrane were imaged under an inverted microscope (200× magnification) followed by manual counting of stained cells.

### 4.9 Sphere Formation Assay

The sphere formation assay was performed as previously reported [60]. Briefly, a density of 4000 cells/well of tumor cells was plated in ultralow attachment plates (Corning, Tewksbury, NY, USA) in serum‐free DMEM supplemented with human recombinant epidermal growth factor (20ng/mL) and 1 × B27 supplement (Invitrogen). After 10 days of incubation, tumor spheroids exceeding 50 µm in diameter were counted under microscope.

### 4.10 Western Blot Analysis

Western blot analysis was performed as previously described [61]. Protein samples were resolved by SDS‐PAGE and subsequently transferred onto a PVDF membrane. After blocking with 5% non‐fat milk in TBST, the membranes were incubated with the following primary antibodies: anti‐GAPDH (sc‐365062; Santa Cruz Biotechnology, CA, USA), anti‐E‐cadherin (#3195; Cell Signaling Technology, MA, USA), anti‐Vimentin (#60300‐1; Proteintech), or anti‐FOXQ1 (#AV39755; Sigma‐Aldrich), along with HRP‐conjugated secondary antibodies, followed by visualization using the ECL detection system (Amersham Biosciences, Piscataway, NJ, USA).

### 4.11 Quantitative PCR Assay

Total RNA was extracted using TRIzol reagent (Invitrogen), and then 2 µg of RNA was reverse transcribed into cDNA with M‐MLV Reverse Transcriptase (Promega, Madison, WI, USA). Quantitative PCR (qPCR) was performed using SYBR Green PCR Master Mix (Invitrogen). Housekeeping gene GAPDH served as the endogenous control for normalization. All primer sequences used in this study are provided in Table S2.

### 4.12 Chromatin Immunoprecipitation Sequencing and qPCR Assays

ChIP was conducted following the manufacturer's protocol (ChIP Assay Kit, Beyotime, Shanghai, China). The experimental procedure consisted of the following steps: (1) Protein‐DNA crosslinking in CRC cells expressing either HOXD4‐Flag or EGFP control using 1% formaldehyde, with subsequent quenching by glycine solution; (2) Chromatin shearing via sonication to generate DNA fragments (200 bp to 1000 bp), which were purified using a DNA purification kit (Beyotime); (3) Immunoprecipitation overnight at 4°C with anti‐Flag antibody or control IgG coupled to Protein A/G beads; (4) Sequential washing with low‐salt, high‐salt, LiCl wash buffer, and TE buffer; (5) Complex elution and crosslink reversal through overnight incubation at 65°C with Proteinase K; (6) DNA purification using a DNA purification kit (Beyotime). The precipitated DNA was subjected to either next‐generation sequencing or qPCR assays (qPCR primer sequences provided in Table S2). Bioinformatics analysis of HOXD4 binding sites and motif identification was performed using the DREME algorithm [62].

### 4.13 RNA Sequencing

Total RNA was extracted from HOXD4‐overexpressing and vector‐control SW620 cells using TRIzol reagent. RNA quality and integrity were assessed prior to library construction. RNA‐seq libraries were prepared and sequenced on the Illumina platform by BGI Genomics. Raw sequencing reads were filtered to remove adaptor sequences and low‐quality reads. Clean reads were aligned to the human reference genome (hg38) using HISAT2. DEGs were identified using DESeq2 with thresholds of |log2 fold change| >1 and adjusted *P* < 0.05. GO enrichment analysis of the DEGs was implemented by the GOseq R packages based Wallenius non‐central hyper‐geometric distribution, which can adjust for gene length bias in DEGs.

### 4.14 Assay for Transposase‐Accessible Chromatin with High‐Throughput Sequencing

ATAC‐seq was performed in HOXD4‐overexpressing and vector‐control HEK293T cells. Briefly, 50 000 cells were harvested by centrifugation at 500 × g for 5 min at 4°C, washed once with cold PBS, and resuspended in cold lysis buffer. Nuclei were isolated by centrifugation at 500 × g for 10 min at 4°C and subsequently resuspended in a transposition reaction mixture containing Tn5 transposase. Following incubation at 37°C for 30 min, transposed DNA fragments were purified. Sequencing libraries were generated by PCR amplification of the purified DNA, purified again, and sequenced on the Illumina platform. Raw sequencing reads were trimmed and aligned to the human reference genome (hg38) using Cutadapt and Bowtie2, respectively. Accessible chromatin regions were identified using MACS2. Differentially accessible regions were analyzed using DiffBind and DESeq2. Peak annotation and functional enrichment analyses were performed using ChIPseeker and clusterProfiler. Motif enrichment analysis was conducted using MEME‐ChIP.

### 4.15 Reporter Constructs and Luciferase Reporter Assay

The FOXQ1 promoter and luciferase gene were cloned into the pLKO.1‐TRC vector (Sigma) using PpuMI and EcoRI restriction enzymes, with concurrent deletion of the vector's U6 promoter. On this basis, a mutant FOXQ1 promoter reporter plasmid lacking the region from ‐153 to ‐92 bp relative to the TSS was constructed using a site‐directed mutagenesis kit (Sigma). Both reporter constructs (Figure S1) were packaged into lentiviruses in 293T cells, which were then used to establish CRC cell lines containing the luciferase reporter. After HOXD4 cDNA and siRNAs were transfected into reporter cells, luciferase activity was measured using the Luciferase Reporter Assay System (Promega) following the manufacturer's protocol.

## Conflicts of Interest

The authors declare no conflicts of interest.

## Supporting information




**Supporting File**: advs76164‐sup‐0001‐SuppMat.docx.

## Data Availability

The data that support the findings of this study are available from [Research Data Deposit]. Restrictions apply to the availability of these data, which were used under license for this study. Data are available from the authors with the permission of [Research Data Deposit].

## References

[1] F. Bray , M. Laversanne , H. Sung , et al., “Global Cancer Statistics 2022: GLOBOCAN Estimates of Incidence and Mortality Worldwide for 36 Cancers in 185 Countries,” CA: A Cancer Journal for Clinicians 74 (2024): 229–263, 10.3322/caac.21834. 38572751

[2] Q. L. Liu , H. Zhou , Z. G. Zhou , and H. N. Chen , “Colorectal Cancer Liver Metastasis: Genomic Evolution and Crosstalk with the Liver Microenvironment,” Cancer and Metastasis Reviews 42 (2023): 575–587, 10.1007/s10555-023-10107-0. 37061644

[3] L. H. Biller and D. Schrag , “Diagnosis and Treatment of Metastatic Colorectal Cancer,” JAMA 325 (2021): 669–685, 10.1001/jama.2021.0106. 33591350

[4] R. Wang , J. Li , X. Zhou , et al., “Single‐cell Genomic and Transcriptomic Landscapes of Primary and Metastatic Colorectal Cancer Tumors,” Genome Medicine 14 (2022): 93, 10.1186/s13073-022-01093-z. 35974387 PMC9380328

[5] S. Gerstberger , Q. Jiang , and K. M. Ganesh , “Metastasis,” Cell 186 (2023): 1564–1579, 10.1016/j.cell.2023.03.003. 37059065 PMC10511214

[6] E. Powell , D. Piwnica‐Worms , and H. Piwnica‐Worms , “Contribution of p53 to Metastasis,” Cancer Discovery 4 (2014): 405–414, 10.1158/2159-8290.Cd-13-0136. 24658082 PMC4063123

[7] T. Y. Na , L. Schecterson , A. M. Mendonsa , and B. M. Gumbiner , “The Functional Activity of E‐Cadherin Controls Tumor Cell Metastasis at Multiple Steps,” Proceedings of the National Academy of Sciences 117 (2020): 5931–5937, 10.1073/pnas.1918167117. PMC7084067 32127478

[8] K. W. Lee , S. Lim , and K. D. Kim , “The Function of N‐Myc Downstream‐Regulated Gene 2 (NDRG2) as a Negative Regulator in Tumor Cell Metastasis,” International Journal of Molecular Sciences 23 (2022): 9365, 10.3390/ijms23169365. 36012631 PMC9408851

[9] C. Megino‐Luque and J. J. Bravo‐Cordero , “Metastasis Suppressor Genes and Their Role in the Tumor Microenvironment,” Cancer and Metastasis Reviews 42 (2023): 1147–1154, 10.1007/s10555-023-10155-6. 37982987 PMC10842895

[10] I. Khan and P. S. Steeg , “Metastasis Suppressors: Functional Pathways,” Laboratory Investigation 98 (2018): 198–210, 10.1038/labinvest.2017.104. 28967874 PMC6545599

[11] L. J. Stafford , K. S. Vaidya , and D. R. Welch , “Metastasis Suppressors Genes in Cancer,” The International Journal of Biochemistry & Cell Biology 40 (2008): 874–891, 10.1016/j.biocel.2007.12.016. 18280770

[12] H. Wang , B. Liu , and J. Wei , “Beta2‐microglobulin(B2M) in Cancer Immunotherapies: Biological Function, Resistance and Remedy,” Cancer Letters 517 (2021): 96–104, 10.1016/j.canlet.2021.06.008. 34129878

[13] D. Y. Torrejon , M. Galvez , G. Abril‐Rodriguez , et al., “Antitumor Immune Responses in B2M‐Deficient Cancers,” Cancer Immunology Research 11 (2023): 1642–1655, 10.1158/2326-6066.Cir-23-0139. 37801341 PMC10842455

[14] Z. Snahnicanova , I. Kasubova , M. Kalman , et al., “Genetic and Epigenetic Analysis of the Beta‐2‐Microglobulin Gene in Microsatellite Instable Colorectal Cancer,” Clinical and Experimental Medicine 20 (2020): 87–95, 10.1007/s10238-019-00601-7. 31853669

[15] J. Janikovits , M. Müller , J. Krzykalla , et al., “High Numbers of PDCD1 (PD‐1)‐Positive T Cells and B2M Mutations in Microsatellite‐Unstable Colorectal Cancer,” Oncoimmunology 7 (2018): 1390640, 10.1080/2162402x.2017.1390640. PMC5749658 29308317

[16] F. Liu , F. Zhong , H. Wu , et al., “Prevalence and Associations of Beta2‐Microglobulin Mutations in MSI‐H/dMMR Cancers,” The Oncologist 28 (2023): e136–e144, 10.1093/oncolo/oyac268. 36724040 PMC10020813

[17] J. Sabates‐Bellver , L. G. Van der Flier , M. de Palo , et al., “Transcriptome Profile of Human Colorectal Adenomas,” Molecular Cancer Research 5 (2007): 1263–1275, 10.1158/1541-7786.Mcr-07-0267. 18171984

[18] Y. Hong , T. Downey , K. W. Eu , P. K. Koh , and P. Y. Cheah , “A ‘Metastasis‐Prone’ signature for Early‐Stage Mismatch‐repair Proficient Sporadic Colorectal Cancer Patients and Its Implications for Possible Therapeutics,” Clinical & Experimental Metastasis 27 (2010): 83–90, 10.1007/s10585-010-9305-4. 20143136

[19] S. Tsuji , Y. Midorikawa , T. Takahashi , et al., “Potential Responders to FOLFOX Therapy for Colorectal Cancer by Random Forests Analysis,” British Journal of Cancer 106 (2012): 126–132, 10.1038/bjc.2011.505. 22095227 PMC3251854

[20] S. Zhou , H. Xu , Y. Duan , Q. Tang , H. Huang , and F. Bi , “Survival Mechanisms of Circulating Tumor Cells and Their Implications for Cancer Treatment,” Cancer and Metastasis Reviews 43 (2024): 941–957, 10.1007/s10555-024-10178-7. 38436892

[21] Y. Huang , W. Hong , and X. Wei , “The Molecular Mechanisms and Therapeutic Strategies of EMT in Tumor Progression and Metastasis,” Journal of Hematology & Oncology 15 (2022): 129, 10.1186/s13045-022-01347-8. 36076302 PMC9461252

[22] W. Lu and Y. Kang , “Epithelial‐Mesenchymal Plasticity in Cancer Progression and Metastasis,” Developmental Cell 49 (2019): 361–374, 10.1016/j.devcel.2019.04.010. 31063755 PMC6506183

[23] S. Koch , “The Transcription Factor FOXQ1 in Cancer,” Cancer and Metastasis Reviews 44 (2025): 22, 10.1007/s10555-025-10240-y. 39777582 PMC11711781

[24] M. Yu , J. Zhan , and H. Zhang , “HOX family Transcription Factors: Related Signaling Pathways and Post‐translational Modifications in Cancer,” Cellular Signalling 66 (2020): 109469, 10.1016/j.cellsig.2019.109469. 31733300

[25] C. Yadav , R. Yadav , S. Nanda , S. Ranga , P. Ahuja , and M. Tanwar , “Role of HOX Genes in Cancer Progression and Their Therapeutical Aspects,” Gene 919 (2024): 148501, 10.1016/j.gene.2024.148501. 38670395

[26] S. A. Jasim , S. H. Farhan , I. Ahmad , et al., “Role of Homeobox Genes in Cancer: Immune System Interactions, Long Non‐Coding RNAs, and Tumor Progression,” Molecular Biology Reports 51 (2024): 964, 10.1007/s11033-024-09857-z. 39240390

[27] R. Morgan , K. Hunter , and H. S. Pandha , “Downstream of the HOX Genes: Explaining Conflicting Tumour Suppressor and Oncogenic Functions in Cancer,” International Journal of Cancer 150 (2022): 1919–1932, 10.1002/ijc.33949. 35080776 PMC9304284

[28] Y. Ying , Y. Wang , X. Huang , et al., “Oncogenic HOXB8 Is Driven by MYC‐Regulated Super‐Enhancer and Potentiates Colorectal Cancer Invasiveness via BACH1,” Oncogene 39 (2020): 1004–1017, 10.1038/s41388-019-1013-1. 31591481

[29] B. Yu and X. Guo , “Prognostic Significance of HOXD4 Protein Expression in Human Ovarian Cancers,” Iranian Journal of Basic Medical Sciences 24 (2021): 1561–1567, 10.22038/ijbms.2021.58396.12969. 35317110 PMC8917843

[30] H. Liu , H. Tian , J. Zhao , and Y. Jia , “High HOXD4 Protein Expression in Gastric Adenocarcinoma Tissues Indicates Unfavorable Clinical Outcomes,” Saudi Journal of Gastroenterology 25 (2019): 46–54, 10.4103/sjg.SJG_105_18. 30588951 PMC6373212

[31] X.‐W. Zhao , Y.‐B. Zhan , J.‐J. Bao , et al., “Clinicopathological Analysis of HOXD4 Expression in Diffuse Gliomas and Its Correlation with IDH Mutations and 1p/19q Co‐Deletion,” Oncotarget 8 (2017): 115657–115666, 10.18632/oncotarget.23371. 29383189 PMC5777801

[32] E. Deforzh , E. J. Uhlmann , E. Das , et al., “Promoter and Enhancer RNAs Regulate Chromatin Reorganization and Activation of miR‐10b/HOXD Locus, and Neoplastic Transformation in Glioma,” Molecular Cell 82 (2022): 1894–1908.e5, 10.1016/j.molcel.2022.03.018. 35390275 PMC9271318

[33] A. W. Lambert , D. R. Pattabiraman , and R. A. Weinberg , “Emerging Biological Principles of Metastasis,” Cell 168 (2017): 670–691, 10.1016/j.cell.2016.11.037. 28187288 PMC5308465

[34] S. U. Khan , K. Fatima , F. Malik , H. Kalkavan , and A. Wani , “Cancer Metastasis: Molecular Mechanisms and Clinical Perspectives,” Pharmacology & Therapeutics 250 (2023): 108522, 10.1016/j.pharmthera.2023.108522. 37661054

[35] Z. Tan , W. Sun , Y. Li , et al., “Current Progress of EMT: A New Direction of Targeted Therapy for Colorectal Cancer with Invasion and Metastasis,” Biomolecules 12 (2022): 1723, 10.3390/biom12121723. 36551152 PMC9775097

[36] H. D. Huh , Y. Sub , J. Oh , et al., “Reprogramming Anchorage Dependency by Adherent‐to‐Suspension Transition Promotes Metastatic Dissemination,” Molecular Cancer 22 (2023): 63, 10.1186/s12943-023-01753-7. 36991428 PMC10061822

[37] S. Usman , N. H. Waseem , T. K. N. Nguyen , et al., “Vimentin Is at the Heart of Epithelial Mesenchymal Transition (EMT) Mediated Metastasis,” Cancers (Basel) 13 (2021): 4985, 10.3390/cancers13194985. 34638469 PMC8507690

[38] J. Yang , P. Antin , G. Berx , et al., “Guidelines and Definitions for Research on Epithelial–Mesenchymal Transition,” Nature Reviews Molecular Cell Biology 21 (2020): 341–352, 10.1038/s41580-020-0237-9. 32300252 PMC7250738

[39] R. Virtakoivu , A. Mai , E. Mattila , et al., “Vimentin–ERK Signaling Uncouples Slug Gene Regulatory Function,” Cancer Research 75 (2015): 2349–2362, 10.1158/0008-5472.Can-14-2842. 25855378

[40] Y. Wang , J. Liu , X. Ying , P. C. Lin , and B. P. Zhou , “Twist‐mediated Epithelial‐mesenchymal Transition Promotes Breast Tumor Cell Invasion via Inhibition of Hippo Pathway,” Scientific Reports 6 (2016): 24606, 10.1038/srep24606. 27094683 PMC4837350

[41] N. Feldker , F. Ferrazzi , H. Schuhwerk , et al., “Genome‐Wide Cooperation of EMT Transcription Factor ZEB1 with YAP and AP‐1 in Breast Cancer,” The EMBO Journal 39 (2020): 103209, 10.15252/embj.2019103209. PMC7459422 32692442

[42] A. Tabatabaee , B. Nafari , A. Farhang , et al., “Targeting vimentin: a Multifaceted Approach to Combatting Cancer Metastasis and Drug Resistance,” Cancer and Metastasis Reviews 43 (2024): 363–377, 10.1007/s10555-023-10154-7. 38012357

[43] A. Jayachandran , B. Dhungel , and J. C. Steel , “Epithelial‐to‐mesenchymal Plasticity of Cancer Stem Cells: Therapeutic Targets in Hepatocellular Carcinoma,” Journal of Hematology & Oncology 9 (2016): 74, 10.1186/s13045-016-0307-9. 27578206 PMC5006452

[44] E. M. Grasset , M. Dunworth , G. Sharma , et al., “Triple‐negative Breast Cancer Metastasis Involves Complex Epithelial‐mesenchymal Transition Dynamics and Requires Vimentin,” Science Translational Medicine 14 (2022): abn7571, 10.1126/scitranslmed.abn7571. PMC9801390 35921474

[45] A. V. Mitchell , L. Wu , C. James Block , et al., “FOXQ1 recruits the MLL Complex to Activate Transcription of EMT and Promote Breast Cancer Metastasis,” Nature Communications 13 (2022): 6548, 10.1038/s41467-022-34239-z. PMC9626503 36319643

[46] C. Wu , C. Zheng , S. Chen , et al., “FOXQ1 promotes Pancreatic Cancer Cell Proliferation, Tumor Stemness, Invasion and Metastasis through Regulation of LDHA‐mediated Aerobic Glycolysis,” Cell Death & Disease 14 (2023): 699, 10.1038/s41419-023-06207-y. 37875474 PMC10598070

[47] M. Yang , Q. Liu , M. Dai , et al., “FOXQ1‐Mediated SIRT1 Upregulation Enhances Stemness and Radio‐Resistance of Colorectal Cancer Cells and Restores Intestinal Microbiota Function by Promoting β‐catenin Nuclear Translocation,” Journal of Experimental & Clinical Cancer Research 41 (2022): 70, 10.1186/s13046-021-02239-4. 35183223 PMC8857837

[48] Y. Qiao , L. Liu , L. Yin , et al., “Retraction Note: FABP4 Contributes to Renal Interstitial Fibrosis via Mediating Inflammation and Lipid Metabolism,” Cell Death & Disease 12 (2021): 801, 10.1038/s41419-021-04084-x. 34417440 PMC8379209

[49] X. Zhang , X. Huang , J. Xu , et al., “NEK2 Inhibition Triggers Anti‐Pancreatic Cancer Immunity by Targeting PD‐L1,” Nature Communications 12 (2021): 4536, 10.1038/s41467-021-24769-3. PMC8316469 34315872

[50] B. Cain and B. Gebelein , “Mechanisms Underlying Hox‐Mediated Transcriptional Outcomes,” Frontiers in Cell and Developmental Biology 9 (2021): 787339, 10.3389/fcell.2021.787339. 34869389 PMC8635045

[51] I. H. Gelman , “Metastasis Suppressor Genes in Clinical Practice: Are They Druggable?,” Cancer and Metastasis Reviews 42 (2023): 1169–1188, 10.1007/s10555-023-10135-w. 37749308 PMC11629483

[52] I. Khan and P. S. Steeg , “A Perspective on the Metastasis Suppressor Field,” Cancer and Metastasis Reviews 42 (2023): 1061–1063, 10.1007/s10555-023-10131-0. 37581870

[53] S. C. Smith and D. Theodorescu , “Learning Therapeutic Lessons from Metastasis Suppressor Proteins,” Nature Reviews Cancer 9 (2009): 253–264, 10.1038/nrc2594. 19242414 PMC2881216

[54] W. Shi , Z. Ye , L. Zhuang , et al., “Olfactomedin 1 Negatively Regulates NF‐κB Signalling and Suppresses the Growth and Metastasis of Colorectal Cancer Cells,” The Journal of Pathology 240 (2016): 352–365, 10.1002/path.4784. 27555280

[55] Q.‐H. Zhou , H. Han , J.‐B. Lu , et al., “Up‐Regulation of Indoleamine 2,3‐Dioxygenase 1 (IDO1) Expression and Catalytic Activity Is Associated with Immunosuppression and Poor Prognosis in Penile Squamous Cell Carcinoma Patients,” Cancer Communications 40 (2020): 3–15, 10.1002/cac2.12001. 32125093 PMC7163927

[56] Y. Li , Z. Ye , S. Chen , et al., “A RHGEF 19 Interacts with BRAF to Activate MAPK Signaling during the Tumorigenesis of Non‐Small Cell Lung Cancer,” International Journal of Cancer 142 (2018): 1379–1391, 10.1002/ijc.31169. 29164615

[57] N. E. Sanjana , O. Shalem , and F. Zhang , “Improved Vectors and Genome‐Wide Libraries for CRISPR Screening,” Nature Methods 11 (2014): 783–784, 10.1038/nmeth.3047. 25075903 PMC4486245

[58] W.‐Y. Du , Z.‐H. Lu , W. Ye , et al., “The Loss‐of‐Function Mutations and Down‐Regulated Expression of ASB3 Gene Promote the Growth and Metastasis of Colorectal Cancer Cells,” Chinese Journal of Cancer 36 (2017): 11, 10.1186/s40880-017-0180-0. 28088228 PMC5237493

[59] T. J. Collins , “ImageJ for Microscopy,” Biotechniques 43 (2007): 25–30, 10.2144/000112517. 17936939

[60] X.‐X. Yuan , Y.‐F. Duan , C. Luo , et al., “Disulfiram Enhances Cisplatin Cytotoxicity by Forming a Novel Platinum Chelate Pt(DDTC)3+,” Biochemical Pharmacology 211 (2023): 115498, 10.1016/j.bcp.2023.115498. 36913990

[61] T. Liu , X. Yue , X. Chen , et al., “Nilotinib in Combination with Sunitinib Renders MCL‐1 for Degradation and Activates Autophagy That Overcomes Sunitinib Resistance in Renal Cell Carcinoma,” Cellular Oncology 47 (2024): 1277–1294, 10.1007/s13402-024-00927-9. 38393513 PMC12974000

[62] T. L. Bailey , “DREME: Motif Discovery in Transcription Factor ChIP‐seq Data,” Bioinformatics 27 (2011): 1653–1659, 10.1093/bioinformatics/btr261. 21543442 PMC3106199

