# Supplementary material for: Genome‐Wide In Vivo RNAi Screening Identifies HOXD4 as a Tumor Metastasis Suppressor in Colorectal Cancer
Source: Adv Sci (Weinh). 2026 Jun 26:e20829. Online ahead of print. doi: 10.1002/advs.202520829 (PMC13336836; doi:10.1002/advs.202520829)
Supplement: Supplementary file 1 — Supporting File: advs76164‐sup‐0001‐SuppMat.docx. [file ADVS-9999-e20829-s001.docx]

**Genome-Wide *In Vivo* RNAi Screening Identifies HOXD4 as a Tumor Metastasis Suppressor in Colorectal Cancer**

Zhi-hua Ye,^1,2,#^ Wen-jing Luo,^1,#^ Lu Li,^1,#^ Wen-di Shuai,^1,3,#^ Ling Zhou,^1^ You-fa Duan,^1,4^ Xue Chen,^1^ Jun-kai Zhang,^2,*^ Wenlin Huang,^1,5,*^ Ran-yi Liu^1,*^

^1^ State Key Laboratory of Oncology in South China, Guangdong Provincial Clinical Research Center for Cancer，Sun Yat-sen University Cancer Center, Guangzhou, Guangdong 510060, China;

^2^ Department of Medical Oncology Center, Zhongshan City People's Hospital, Zhongshan, Guangdong 528403, China;

^3^ Department of Oncology, Qingdao Municipal Hospital, Qingdao, Shandong 266011, China;

^4^ Guiyang Healthcare Vocational University Clinical and Rehabilitation College, Guiyang, Guizhou 550081, China;

^5^ Guangdong Provincial Key Laboratory of Tumor Targeted Drugs & Guangzhou Enterprise Key Laboratory of Gene Medicine, Guangzhou Doublle Bioproducts Co. Ltd., Guangzhou, Guangdong 510663, China.

#These authors contributed equally to this work.

Correspondence to: Ran-yi Liu (liury@sysucc.org.cn), Wenlin Huang (hwenl@mail.sysu.edu.cn), or Jun-kai Zhang (jkz1103@163.com).

**Table S1: Candidate tumor metastasis suppressor in colorectal cancer by genome-wide *in vivo* screening.**

| **Library** | **No. of mice** | **Mice with liver metastasis** | **No.of genes identified in at least two mice** | **Genes supposed to be downregulated in CRC** |
| --- | --- | --- | --- | --- |
| Pool 1 | 8 | 8 | 6 | DGKA, CKMT1B |
| Pool 2 | 9 | 9 | 6 | GPR82, HOXD4 |
| Pool 3 | 8 | 3 | N/A | N/A |
| Pool 4 | 8 | 5 | N/A | N/A |
| Pool 5 | 8 | 5 | 2 | B2M |
| Pool 6 | 8 | 8 | N/A | N/A |
| Pool 7 | 8 | 2 | N/A | N/A |
| Pool 8 | 5 | 3 | 5 | ZNF518A, FAM177A1 |
| Pool 9 | 8 | 3 | N/A | N/A |
| Pool 10 | 8 | 4 | N/A | N/A |

**Table S2: The information of siRNA, shRNA and sgRNA used in this study.**

| **Name** | **Targeted sequence** |
| --- | --- |
| CKMT1B shRNA | 5'-CGGTGTCTTTGATATTTCTAA-3' |
| DGKA shRNA | 5'-GCTAAATATGTCCAAGGAGAT-3' |
| ZNF518A shRNA | 5'-GCCACCTGAAGTAAACCAATT-3' |
| GPR82 shRNA | 5'-GCTTCCTTCCTTATAGTATTT-3' |
| FAM177A1 shRNA | 5'-CCCGTCATTCATCATTTAGAA-3' |
| HOXD4 shRNA/siRNA-1 | 5'-CCAACACTAAAGGCAGGTCAT-3' |
| HOXD4 siRNA-2 | 5'-GCCCTCGGACTAGGTTAGCAT-3' |
| FOXQ1 siRNA-1 | 5'-CAGGCTTCGTCTTATTTCT-3' |
| FOXQ1 siRNA-2 | 5'-CTCCATCAAACGTGCCTTA-3' |
| HOXD4 sgRNA-1 | 5'-CGCGCCGCCGCCGTAGTAGT-3' |
| HOXD4 sgRNA-2 | 5'-CAGGGCGCCGACTACTACGG-3' |

**Table S3: The primers used for qPCR in this study.**

| **Name** | **Direction** |  |
| --- | --- | --- |
| GAPDH-F | Forward | 5'-AGAAGGCTGGGGCTCATTTG-3' |
| GAPDH-R | Reverse | 5'-AGGGGCCATCCACAGTCTTC-3' |
| HOXD4-F | Forward | 5'-CCCTCCGTGCGAGGAGTAT-3' |
| HOXD4-R | Reverse | 5'-GAAAGGCTGCTCACCGAAGT-3' |
| CKMT1B-F | Forward | 5'-TGAACGACGGAGGCTGTATC-3' |
| CKMT1B-R | Reverse | 5'-CCGTGCATAGACTGCTGGG-3' |
| DGKA-F | Forward | 5'-AGGATGGCGAGATGGCTAAAT-3' |
| DGKA-R | Reverse | 5'-CCAGGCTTAGGTGTCTGGG-3' |
| FAM177A1-F | Forward | 5'-CCCAAGGAGAGTCATCCACTT-3' |
| FAM177A1-R | Reverse | 5'-TCGGATCAACAGTAGGCAAAAC-3' |
| GPR82-F | Forward | 5'-GGGAGTTGTACTGGGCATAATC-3' |
| GPR82-R | Reverse | 5'-TCATGGCTCCTAGTTCCATCTG-3' |
| FOXQ1-F | Forward | 5'-CACGCAGCAAGCCATATACG-3' |
| FOXQ1-R | Reverse | 5'-CGTTGAGCGAAAGGTTGTGG-3' |
| FOXQ1 ChIP-F | Forward | 5'-GTGGCACGCACATCATCCGG-3' |
| FOXQ1 ChIP-R | Reverse | 5'-ACTTTGGGGTGAGTGTGTGCAG -3' |


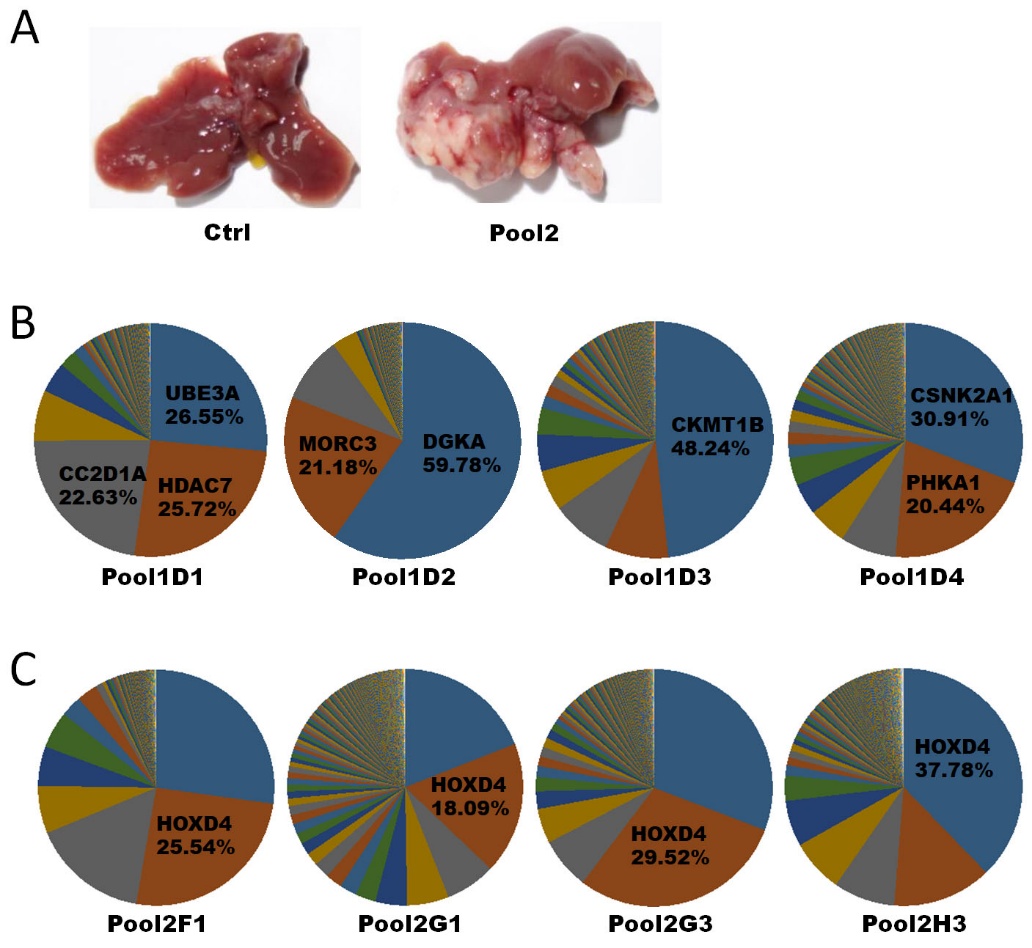


**Figure S1: Genome-wide RNAi screening for metastasis suppressor genes in colorectal cancer.** **A)** Representative gross liver images from experimental (pool2) and control groups at week 5 post-injection. **B)** Next-generation sequencing analysis of shRNA enrichment patterns in 4 metastatic liver nodules from mouse #Pool1D. **C)** Distribution of HOXD4-targeting shRNAs in 4 metastatic nodules across three individual mice (#Pool2F, #Pool2G and #Pool2H).

**
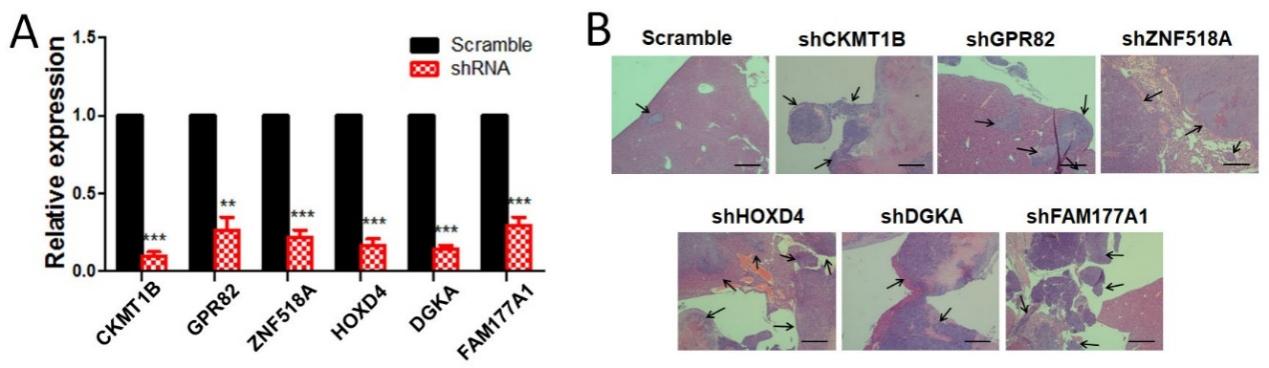
**

**Figure S2: The impact of candidate gene knockdown on the liver metastatic potential of HCT116 cells.‌ ‌A)‌** Knockdown efficiency of candidate genes in HCT116 cells was confirmed by RT-qPCR. **‌B)‌** Representative H&E-stained liver sections showing metastatic foci (indicated by arrows) from mice intrasplenically injected with gene-knockdown HCT116 cells.

**
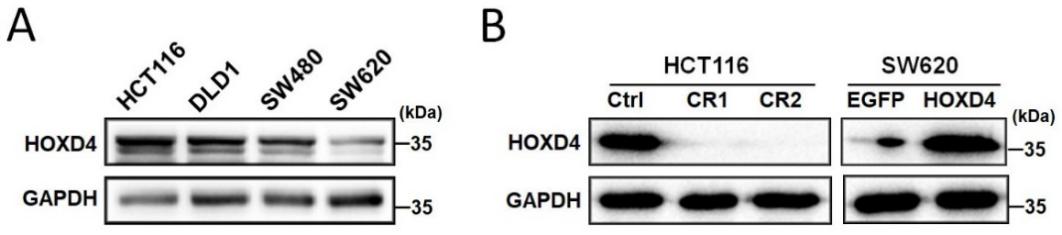
**

**Figure S3: Establishment of stable HOXD4-modified colorectal cancer cell lines. ‌A)**‌ Western blot analysis of basal HOXD4 expression levels in colorectal cancer cell lines‌. **‌B)‌** Western blot validation of genetic modification efficiency: HOXD4 knockout in HCT116 cells (CR1/CR2: independent clones via CRISPR-Cas9; Ctrl: mock-transfected control) versus HOXD4 overexpression in SW620 cells (HOXD4, HOXD4 overexpression; EGFP, EGFP-expression as a negative control).

**
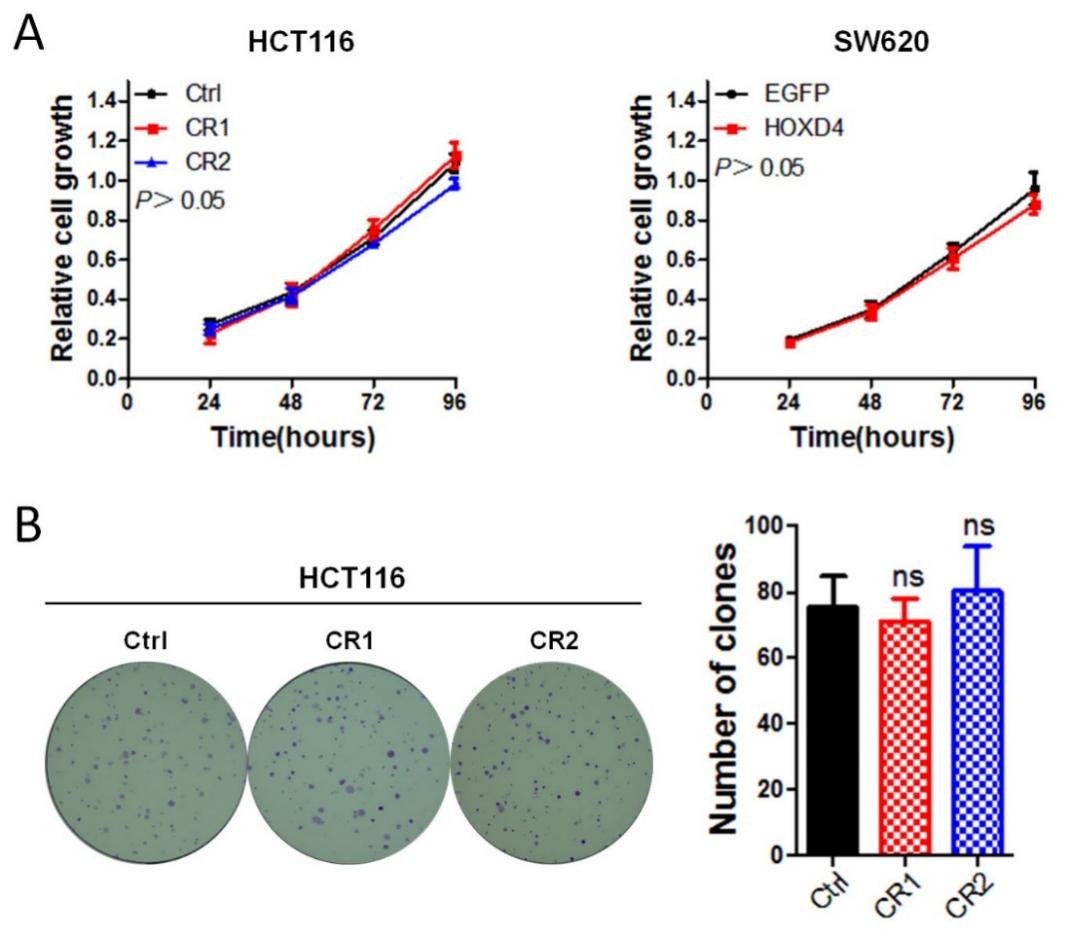
**

**Figure S4: HOXD4 does not influence colorectal cancer cell proliferation. ‌A)** Cell viability assessed by MTT assay in HCT116 cells with HOXD4 knockout (left) and SW620 cells with HOXD4 overexpression (right). **‌B)**‌ Colony formation assays in HCT116 cells with HOXD4 knockout. Left: Representative images of crystal violet-stained colonies; Right: Quantitative analysis of colony numbers. CR1/CR2, HCT116 cells with HOXD4 knockout; Ctrl, HCT116 cells with mock-transfected; HOXD4, SW620 cells with HOXD4 overexpression; EGFP, SW620 cells with EGFP expression as a negative control; ns, not significant (p > 0.05).

**
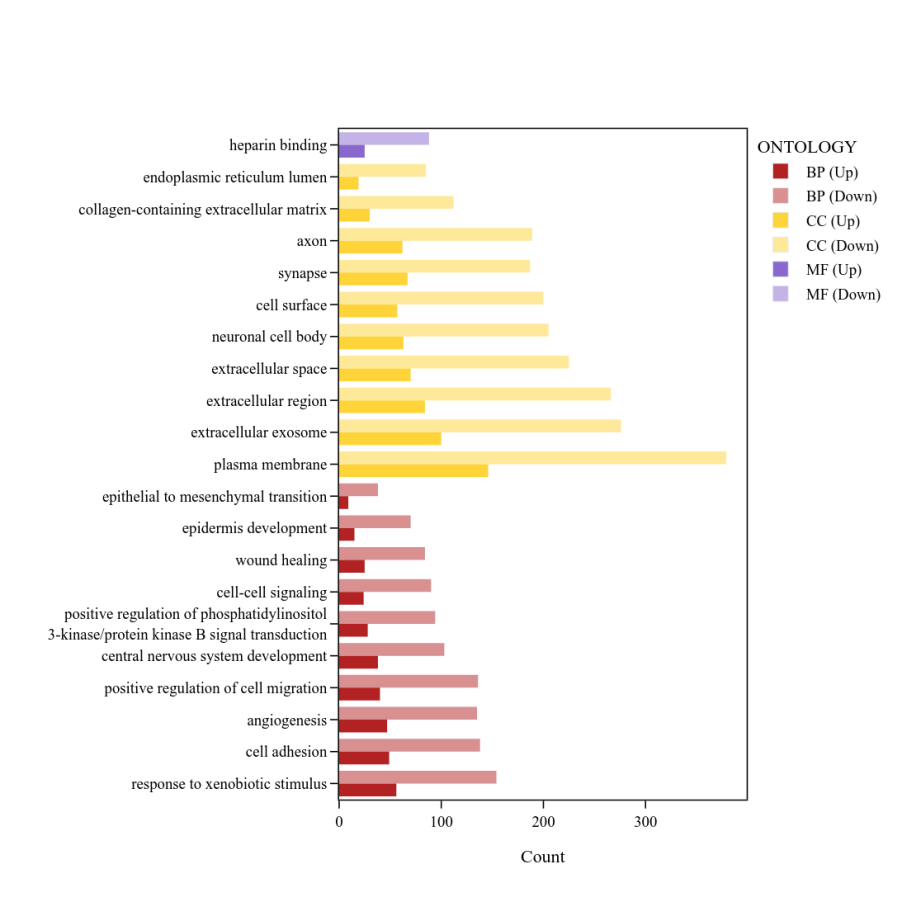
**

**Figure S5: Directional GO enrichment bar plot of transcriptomic changes in HOXD4-overexpressing SW620 cells.** Enrichment analysis was performed separately for upregulated and downregulated gene cohorts.

**
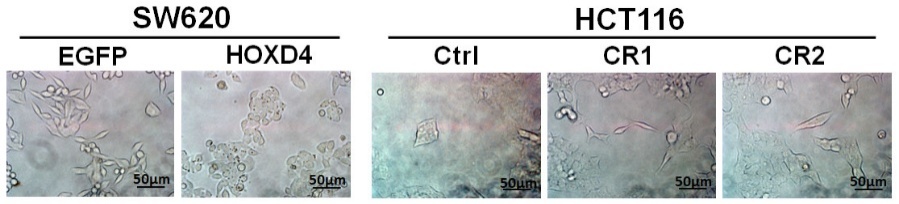
**

**Figure S6: Morphological characterization of HOXD4-modified colorectal cancer cells under high-magnification phase-contrast microscopy.** HOXD4, SW620 cells with HOXD4 overexpression; EGFP, SW620 cells with EGFP expression as a negative control; CR1/CR2, HCT116 cells with HOXD4 knockout; Ctrl, HCT116 cells with mock-transfected.

**
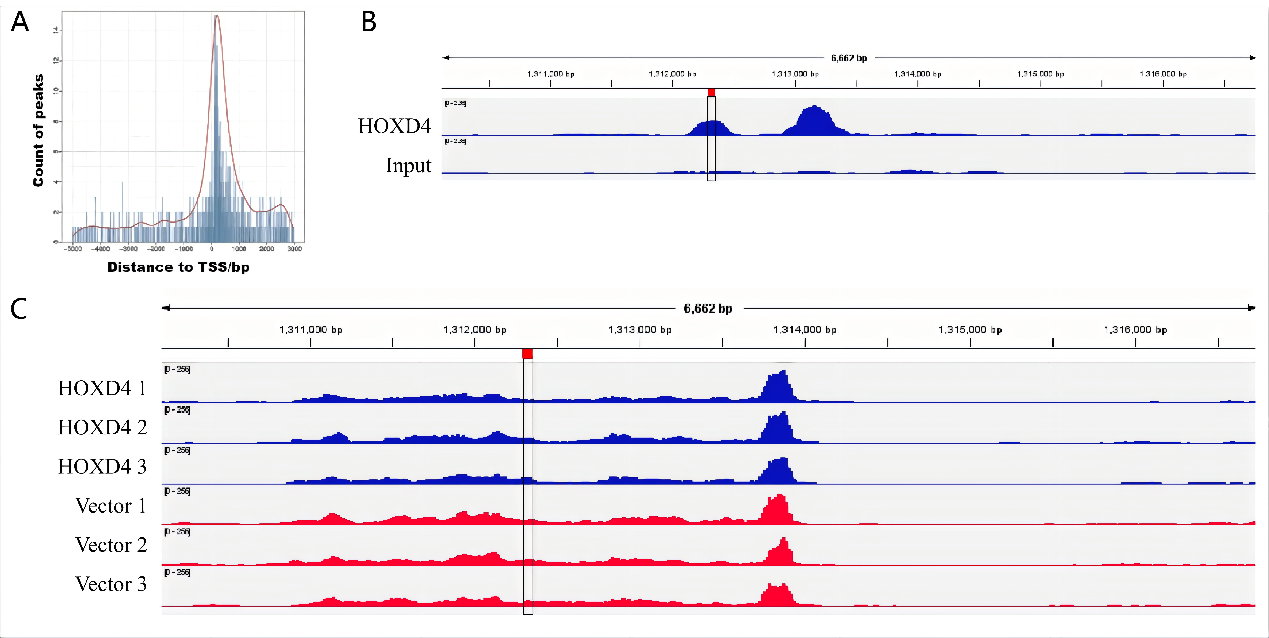
**

**Figure S7: HOXD4 ChIP-seq and ATAC-seq analysis. ‌A)** Genome-wide profiling of HOXD4 binding sites near transcription start sites by ChIP sequencing assay. **‌B)** HOXD4 ChIP-seq signal at the FOXQ1 locus. The boxed region corresponds to the FOXQ1 promoter fragment (-153 to -92 bp). **‌C)** Chromatin accessibility assessed by ATAC-seq at the FOXQ1 locus. The boxed region corresponds to the FOXQ1 promoter fragment (-153 to -92 bp).


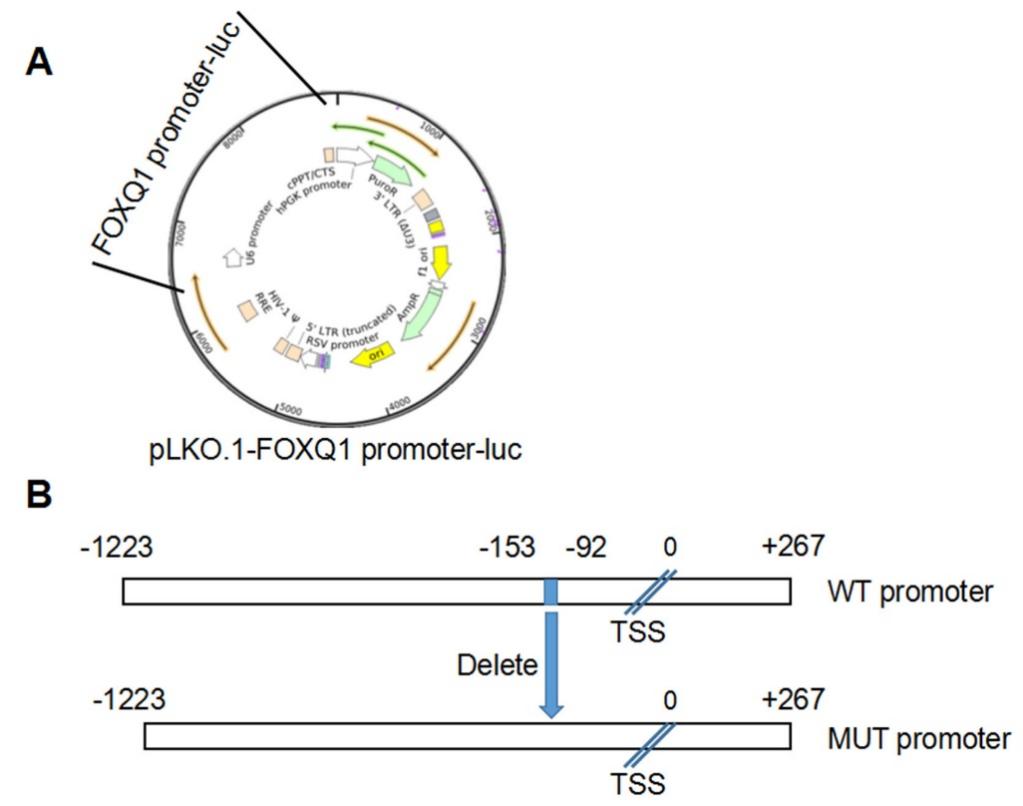


**Figure S8:** **Schematic illustration of the FOXQ1 promoter reporter plasmid construction.‌ A)** Overall structure of the reporter plasmid. **B)** Schematic representation of wild-type (WT) and mutant (MUT) FOXQ1 promoters.
